# Comparative biomechanical analysis of monocortical and bicortical polyaxial screw rod fixation in canine lumbar vertebral stabilization

**DOI:** 10.3389/fvets.2024.1434251

**Published:** 2025-02-12

**Authors:** Julien Guevar, Benjamin Voumard, Robert Bergman, Christina Precht, Franck Forterre

**Affiliations:** ^1^Division of Small Animal Surgery, Vetsuisse Faculty, University of Bern, Bern, Switzerland; ^2^ARTORG Centre for Biomedical Engineering Research, University of Bern, Bern, Switzerland; ^3^Synapse Veterinary Neurology, LLC, Charlotte, NC, United States; ^4^Division of Small Animal Radiology, Vetsuisse Faculty, University of Bern, Bern, Switzerland

**Keywords:** lumbar spine stabilization, polyaxial screws, canine neurosurgery, biomechanical evaluation, veterinary orthopedics

## Abstract

**Objective:**

This study aims to evaluate the biomechanical properties of polyaxial screws-rod fixation (PSR) in stabilizing a single vertebral motion unit (VMU) fracture model and to compare the effectiveness of different stabilization techniques such as monocortical and bicortical.

**Methods:**

A total of 12 thoracolumbar vertebral column specimens were harvested from canine cadavers. These specimens were divided into two groups based on the stabilization technique applied: a monocortical group and a bicortical group. Each group underwent biomechanical testing to assess flexion/extension and lateral bending motions. The range of motion (ROM), neutral zone (NZ), and stiffness were measured for each lumbar VMU in three conditions: intact, fractured with unilateral stabilization, and fractured with bilateral stabilization.

**Results:**

In the 3-column fracture model, PSR was unable to restore the ROM of an intact spine in flexion/extension. In lateral bending, only bilateral PSR successfully approached the ROM of the intact spine. Notably, PSR failures were observed in four specimens when applied as monocortical and unilateral stabilization.

**Conclusion:**

The findings indicate that even bilateral PSR does not fully restore the intact spine's ROM in canine fracture models, highlighting the need for further research to optimize stabilization techniques. The current study demonstrates that a single 3-column lumbar fracture model VMU cannot be adequately stabilized using PSR in a canine model, suggesting potential limitations in both monocortical and bicortical approaches.

## 1 Introduction

The canine lumbar spine is a common site for traumatic vertebral fracture/luxation and for vertebral instability of congenital or iatrogenic origin (1). Although the decision on *when* to stabilize the vertebral column appears straightforward (two compartments of the 3-column model), the *how* remains not so clear (2, 3). Despite a few canine studies (4–8), the majority of current stabilization techniques are based on biomechanical assumptions derived from canine long-bone orthopedic principles and/or translated from human spine knowledge. Currently, vertebral stabilization in veterinary surgery lacks a robust evidence-based foundation (9). The stabilization techniques we have used in the past appear to work and have stood the test of time. However, understanding the canine-specific biomechanical properties of both old and new implants can only make constructs stronger and neurosurgery safer.

In the realm of veterinary neurosurgery, human pedicle screw rod fixation (PSR), specifically the polyaxial type, has become a versatile tool akin to a “Swiss knife” for stabilization in dogs. This technology, a relative newcomer in veterinary neurosurgery, has been the gold standard in human surgery for over three decades (10). Unlike traditional plate and screw stabilization, each PSR allows for optimal placement in areas with maximal vertebral bone stock, ensuring robust bone purchase before being interconnected by a rod. This “internal fixator” is made from medical-grade titanium, making it MRI-compatible and thus suitable for postoperative or follow-up imaging.

**Figure 1:**
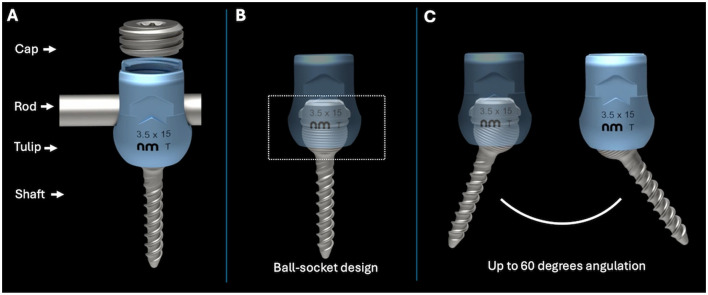
**(A)** The different parts of a polyaxial pedicle screw. **(B)** Design of the polyaxial screw used in this study. **(C)** Degrees of angulation possible with a polyaxial screw using a ball-in-the-socket design.

Compared to screw-and-cement methods, PSR systems are less bulky, carry a reduced risk of infection, and allow for straightforward revisions by disconnecting the three polyaxial screw components: the screw [head (tulip), screw shaft], the rod, and the set screw (or cap; Figure 1) (11, 12). Veterinary polyaxial systems, such as those from (ex: Invetra (Neuromed); Pedro (Overvet), Vesta (Travmavet), Arcas (Artemedics), Fusion Implants pedicle screws systems) have emerged on the market, featuring screw shaft designs closely mirroring their human counterparts (13–17). However, the optimal design for canine vertebrae has not been evaluated, and it is unclear whether designs such as cortical screws, cancellous screws, hybrid cortical/cancellous, or locking screws offer specific advantages for canine spine stabilization.

The research field for spinal stabilization in veterinary medicine remains widely unexplored, and despite some existing knowledge (5, 18), significant uncertainties persist. For instance, it is not clear whether stabilization should be unilateral or bilateral and whether it should employ mono- or bicortical fixation to optimally stabilize the thoracolumbar spine (19). Additionally, the relationship between the type of vertebral fracture/luxation type and the number of vertebral segments requiring stabilization is also not well-defined. Given these gaps, there is a pressing need for comprehensive, foundational research in this area. With this in mind, our study aims to advance the understanding of the biomechanics of pedicle screw fixation in a single vertebral motion unit (VMU), providing essential insights that could inform future developments in canine spinal stabilization.

In this research study, our objective was to evaluate the biomechanical properties of polyaxial screws-rod fixation of a single VMU to stabilize a 3-column fracture model of the canine lumbar spine. With respect to a clinical setting, we wished to understand if the biomechanical properties of the intact spine could be restored using a single VMU stabilization. We aimed to study and compare the ranges of motion (ROM), neutral zone, and stiffness in flexion/extension and lateral bending of a single lumbar VMU in an intact spine and a 3-column “fracture/luxation model” using different stabilization configurations. We hypothesized that (1) a bicortical or monocortical bilateral PSR construct will restore intact spine biomechanical properties in a 3-column fracture model and (2) that a monocortical or bicortical unilateral construct will not.

## 2 Material and methods

### 2.1 Study design

**Figure 2:**
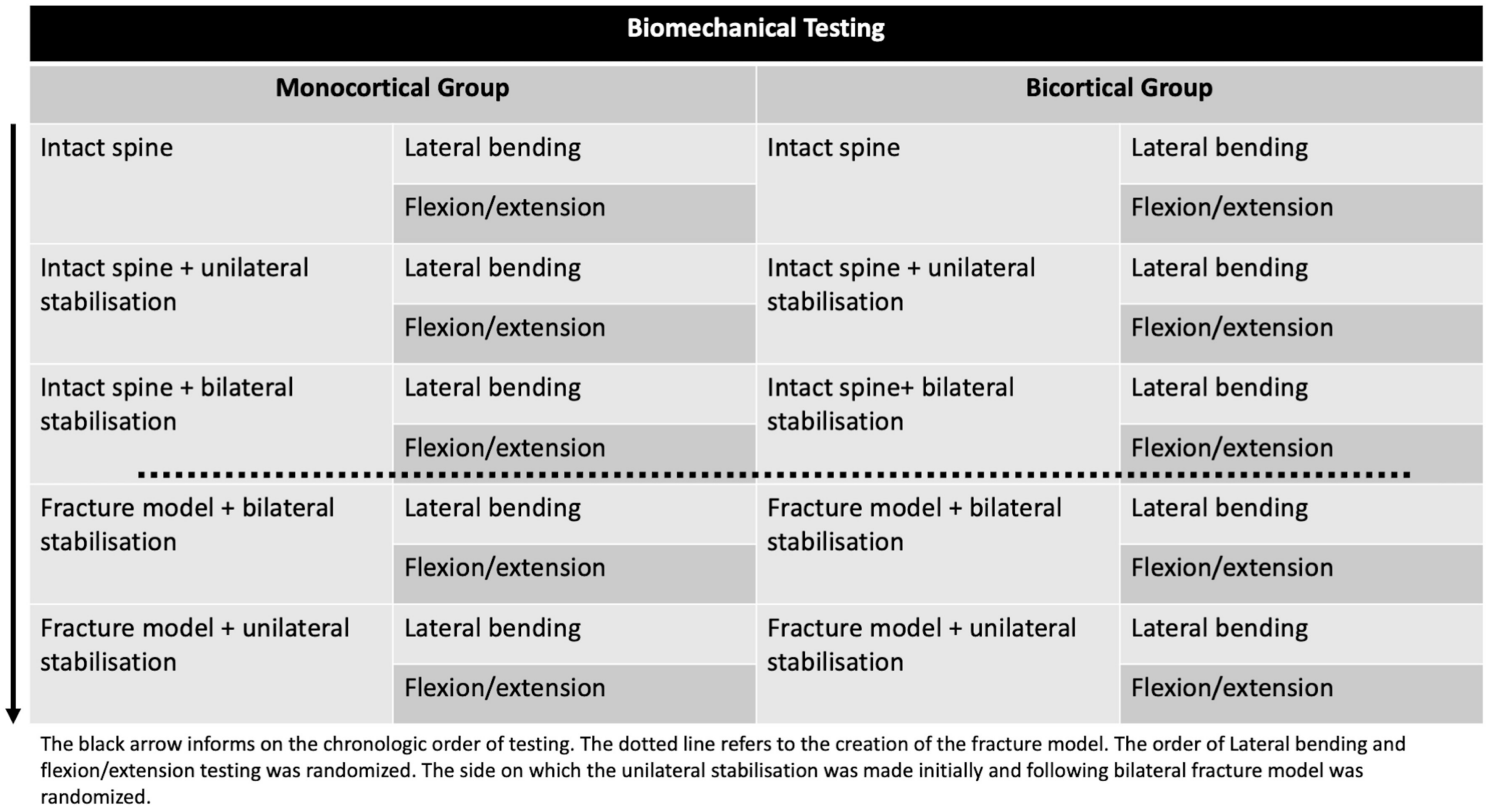
Biomechanical testing flowchart for the two groups (monocortical and bicortical). The intact spine was first tested in flexion/extension and lateral bending. A unilateral PSR was then added (side randomization) and tested. The PSR was then added on the contralateral side and tested. The intervertebral disc, posterior ligament complex, and articular process capsules were removed, creating a 3-column fracture model with bilateral PSR in place, and the model was tested in the two directions. The unilateral PSR construct was then tested following the contralateral side PSR disassembly.

**Figure 3:**
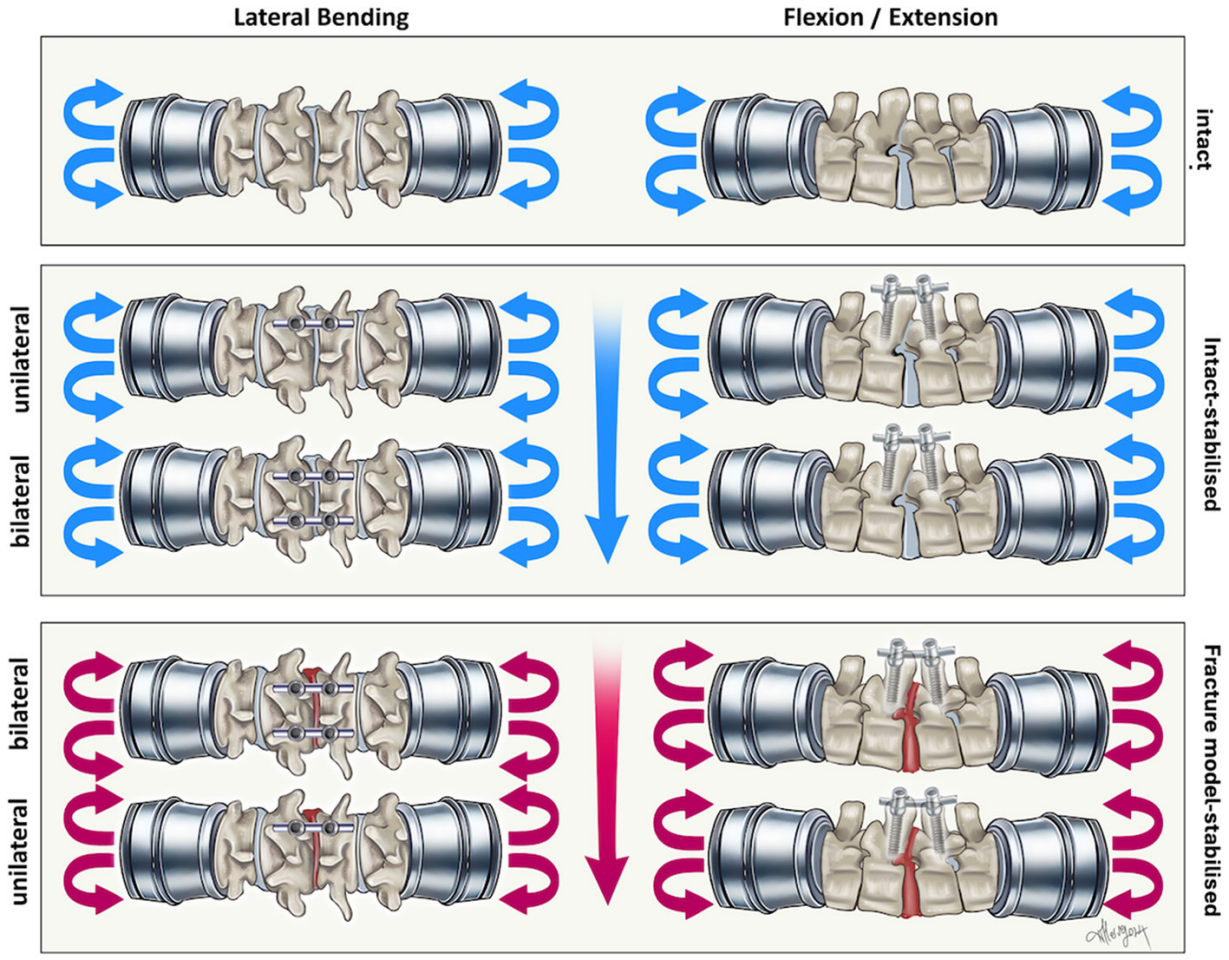
Illustration of the biomechanical testing in the present study as described in Figure 2. Testing was performed in this manner for the monocortical and bicortical group.

The thoracolumbar vertebral column specimens (T13-L3 segments) collected from 12 canine cadavers were assigned to a cortical purchase testing group [monocortical group (*n* = 6) and bicortical group (*n* = 6)]. All T13-L3 segments were tested in flexion/extension and lateral bending. Axial rotation was not tested. Following neutralization of the T13-L1 and L2-L3 VMUs, the L1-L2 VMU was tested for each group in the two directions as (1) intact specimen, (2) with unilateral fixation, (3) with bilateral fixation, then following creation of the fracture model, (4) with bilateral fixation, and finally (5) with unilateral fixation. Figures 2, 3 summarize and illustrate the sequence in which testing was performed. The study was performed in this sequence to minimize the number of cadavers to be used, with the 3R principles of scientific research in mind (20). For the 3-column fracture model (fracture/luxation model), the posterior ligamentous complex (interspinous ligament, dorsal spinous ligament, articular process capsules), dorsal and ventral longitudinal ligaments, and intervertebral disc between L1–L2 were removed. Each unilateral construct comprised two polyaxial pedicle screws and one rod, while bilateral constructs included four screws and two rods. Randomization of the initial stabilization side, the rod removal side for fracture model testing, and the testing sequence (flexion/extension, lateral bending) was achieved using a computer-generated random number table (Excel and Microsoft). Implants were visually confirmed as monocortical or bicortical during testing and verified post-testing using CT imaging.

### 2.2 Specimen collection and preparation

This original research study was considered a sub-threshold for ethical approval by the conveyor of ethics at our institution. Consent to use the canine cadavers had been granted by their previous owners.

Thoracolumbar vertebral specimens (T13-L3) were collected from canine cadavers [median body weight: 12 kg (SD: 1.1)] of a single breed (Beagle). The dogs were skeletally mature and had no evidence of vertebral column disease on CT. All vertebral ligaments (supraspinous and interspinous ligaments and ligamentum flavum), intervertebral discs, and joint capsules were preserved. Epaxial musculature was removed. Tissues were maintained in a moist state using gauze impregnated with 0.9% saline solution throughout preparation, storage, and testing. Specimens were individually wrapped in saline-soaked towels and plastic bags and stored at −20°C.

**Figure 4:**
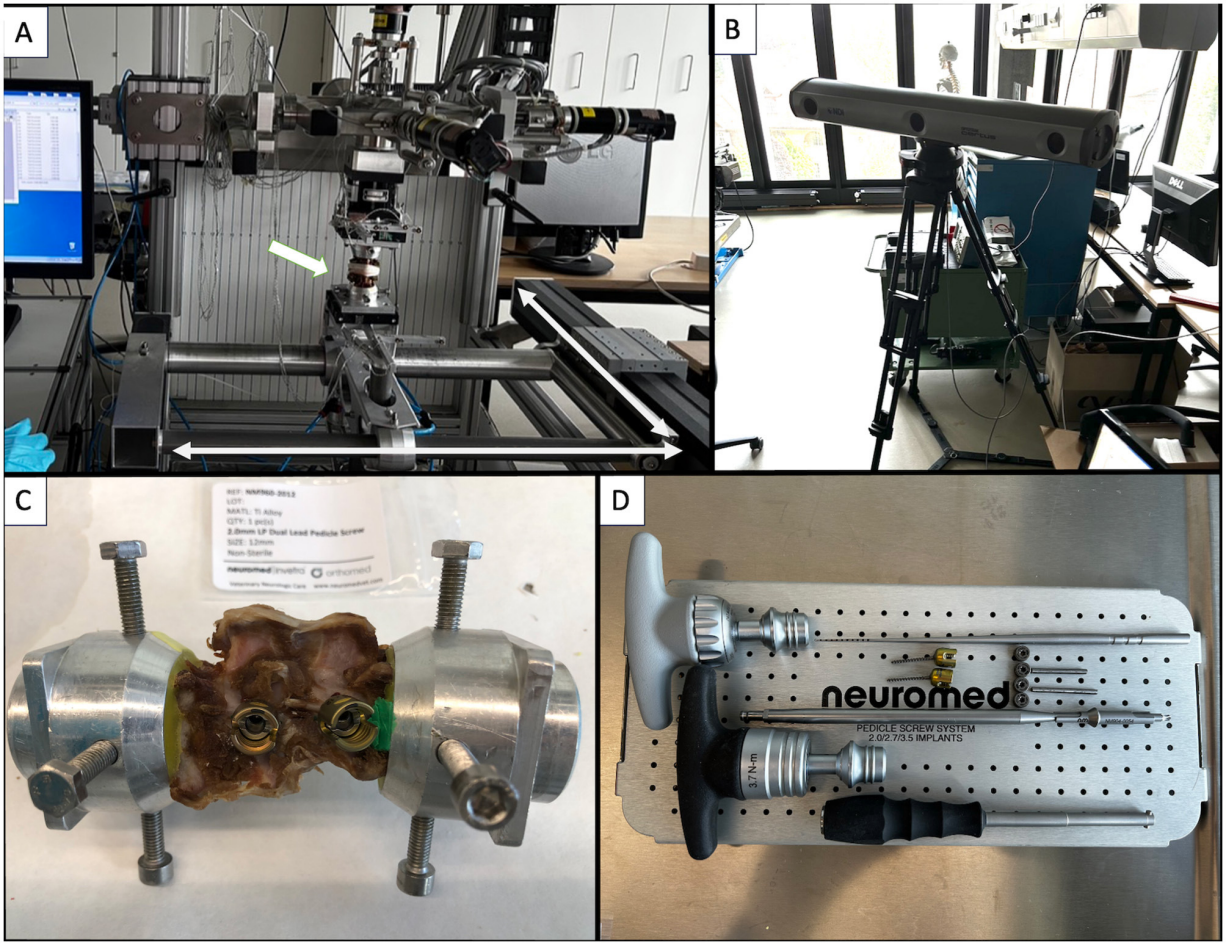
**(A)** Spine tester (arrow: Specimen being tested; bidirectional arrows: directions of motion). **(B)** Infrared camera. **(C)** Spine segment in fixture pots filled with PMMA. **(D)** Neuromed polyaxial pedicle screw kit.

Spine segments were thawed to room temperature on the day of testing and then mounted in a spine tester with infrared motion capture (Figures 4A, B). The T13-L1 and L2-L3 VMUs were neutralized using 2 × 1.6 mm Steinman pins inserted in T13 and L3, traversing the intervertebral disc and penetrating the cranial part of the L1 vertebra and the caudal part of the L2 vertebra. T13 and L3 were cleaned of all soft tissue, and part of the spinous process was removed and secured in fixture pots by using the four spine tester fixture pot screws. The screws, along with the cranial third of T13 and the caudal third of L3, were embedded in polymethylmethacrylate (PMMA) (Technovit, Kulzer, Germany; Figure 4C). Intact specimens allowed motion at a single VMU: L1-L2. T13-L1 and L2-L3 VMUS absence of motion was inspected visually by two observers before testing. These are referred to as the “spine segments.”

**Figure 5:**
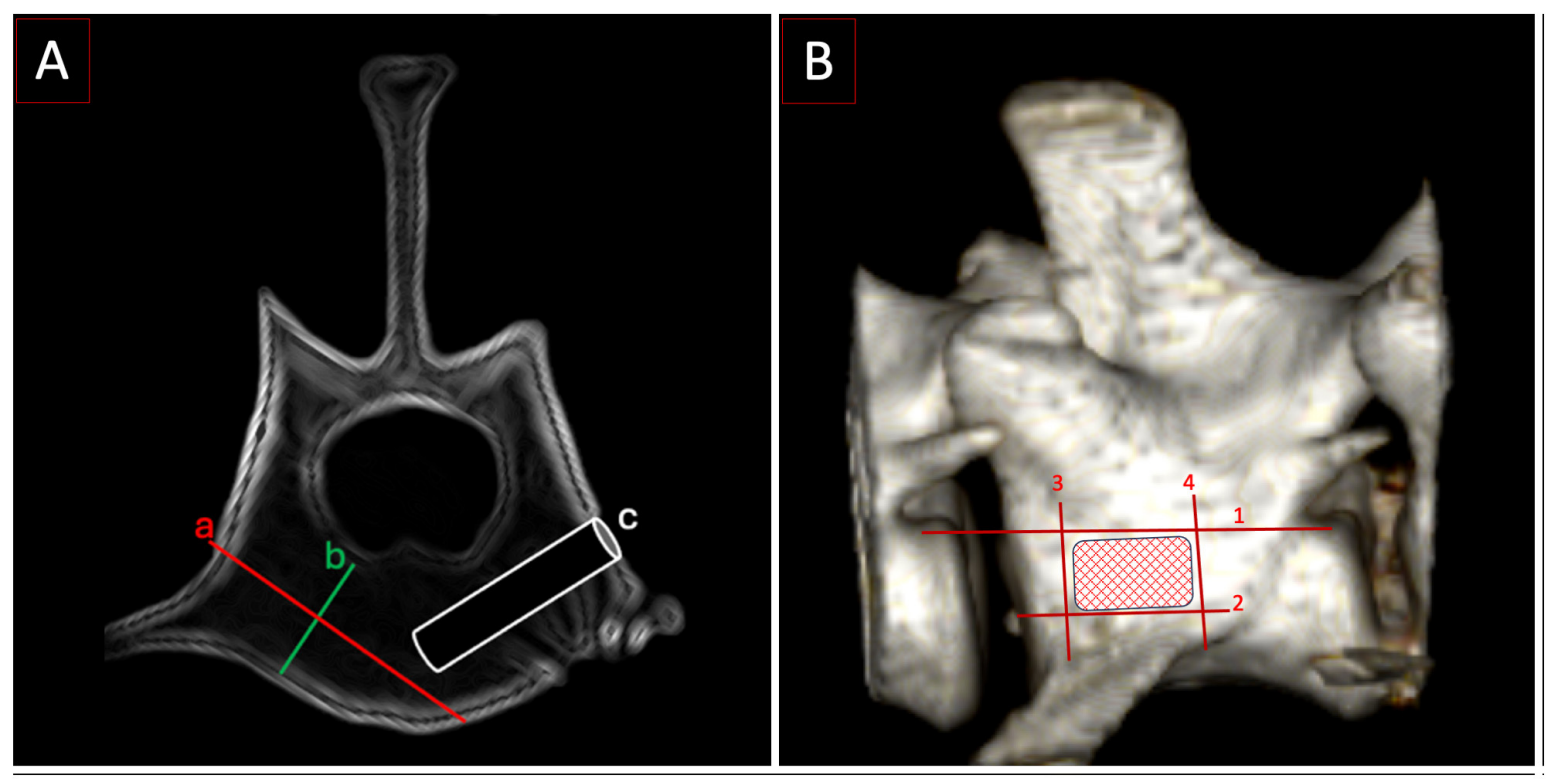
**(A)** Postoperative CT with trajectory and depth for a monocortical screw. a: Distance from cortex to cortex, b: the width of the bone stock in the vertebral body, c: the width of a 2 mm screw is 33% of b. **(B)** Entry point zone for the screws. Lines 1–4 delineate the area of insertion: (1) the floor of the vertebral canal, (2) the base of the transverse process, (3) the cranial edge of the transverse process at its starting point on the vertebral body, and (4) the caudal edge of the transverse process at its starting point on the vertebral body.

Polyaxial pedicle screws (Neuromed, Invetra: diameter: 2.0 mm and lengths: 12 mm for monocortical and 18 mm for bicortical constructs) were inserted following predrilling with a 1.5 mm drill bit (Figure 4D). Screw diameter was established based on preoperative CT in light of the available safe bone corridor of all vertebrae. In the absence of specific guidelines, we estimated that screw size should be between 33%−66% of the bone mass available in the vertebral body (based on the authors' clinical experience). The bone mass available was calculated by measuring the distance (b) between the inner and the outer cortices. The width would have to be perpendicular to the expected trajectory (a) (Figure 5A). Ultimately, in our study, the diameter of the screws (c = 2 mm) was 33% of the bone stock available (b = 6 mm). Using CT data acquired prior to testing, the aforementioned screw lengths were selected to achieve mono- or bicortical purchase of the vertebral body. The screw entry points for each vertebra were approximated to a similar safe entry point based on CT data in a “clinical case” fashion (4). The exit points were such that screw trajectories would not cross paths (Figure 5B). Convergence or divergence of ipsilateral screws was not defined.

### 2.3 Specimen testing

The spine segments were attached to a customized spinal loading simulator with three linear and three rotational degrees of freedom. A torque was applied with electric motors in one axis while the two others were free to rotate, and the three-translation axis was gliding on air bearings. Torque was applied within a range of −2 to +2 Nm at a rate of 1°/s. Two modes were tested: flexion/extension and lateral bending. A load cell (MC3A, AMTI, USA) recorded the torque while positioning markers tracked by a motion capture camera (Certus Optotrak, Northern Digital, Canada) at 100 Hz allowed the computation of the angle between the top and bottom plates, which is the angle of the L1-L2 functional spinal unit. Each cycle was repeated three times to minimize viscoelastic creeping, and the last cycle was reserved for the computation of the results. The synchronization of the torque signal with the angle allowed us to compute the torque-angle diagram to extract the ROM.

The relative ROM of the treated segment in all four stabilization configurations was defined as the percentage of the ROM relative to the ROM of the untreated segment (intact specimen), which was considered to have 100% ROM. Neutral zone and stiffness and relative neutral zone and relative stiffness are parameters provided by the software. The neutral zone (RNZ) refers to the spinal segment's range of motion (ROM) that occurs with minimal resistance. The stiffness quantifies the resistance of the construct to deformation under applied loads and is calculated as the slope of the load-displacement curve.

### 2.4 Statistical analysis

Power analysis was carefully considered prior to testing. Following a thorough literature review (21–25), no “golden number” could be identified for a spine biomechanical study, and therefore, consultation with the biomechanical spine testing laboratory (which has know-how and repeatability of methodology expertise) led to the suggestion that six specimens per group should be sufficient. The Shapiro–Wilk test was used to establish the normality of the distribution, and the Wilcoxon signed-rank test was performed to compare differences between groups in the context of small sample groups. The *P*-value significance was set at < 0.05. Descriptive statistics for central tendency and variability are expressed using the median and the standard deviation or IQR.

## 3 Results

**Figure 6:**
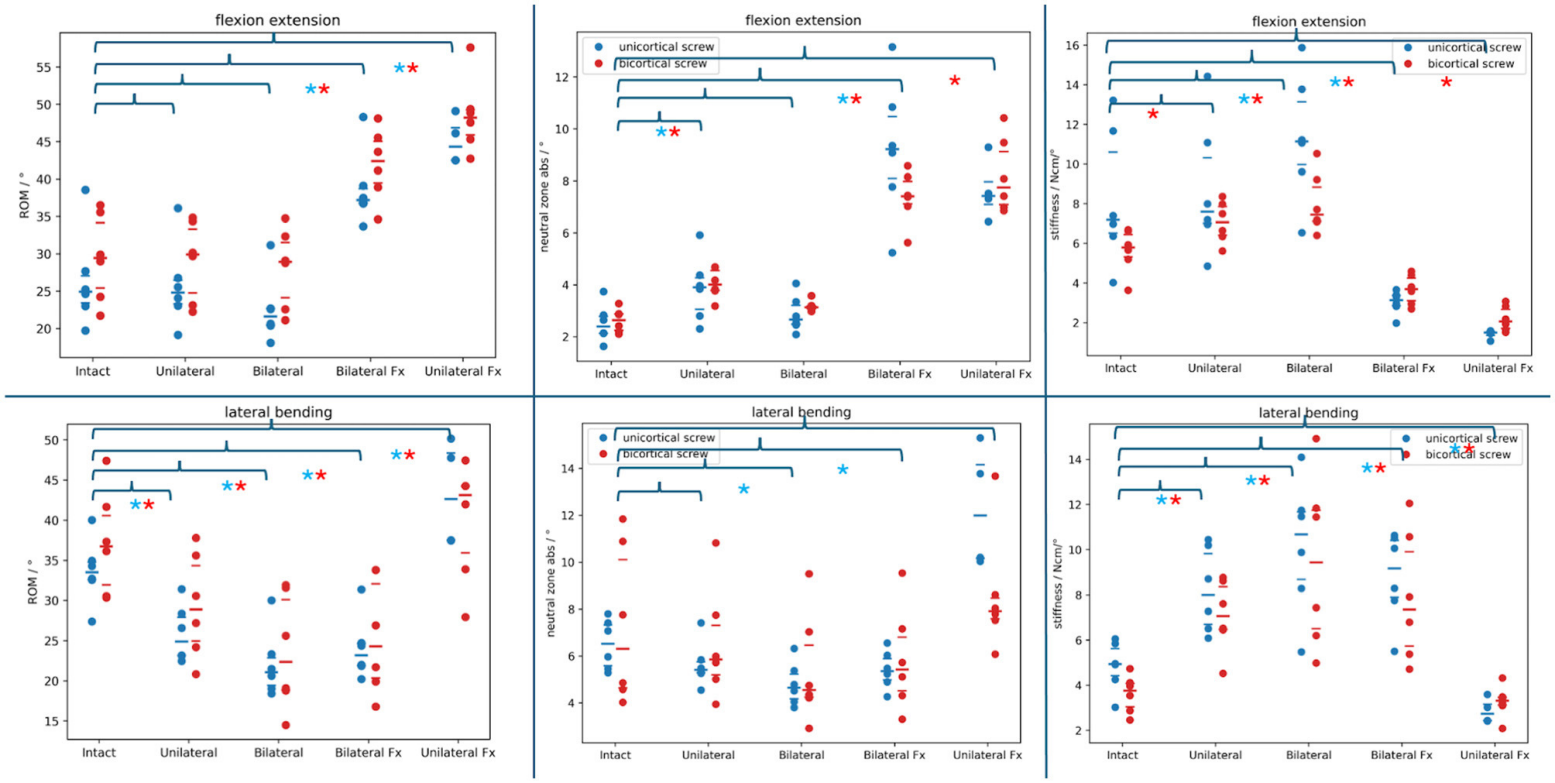
Absolute range of motion, neutral zone, and stiffness datasets of the different PSR constructs compared to the intact spine (brackets above) in flexion/extension and lateral bending. Blue dots: monocortical screws; red dots: bicortical screws. Blue asterisks note a statistically significant difference between the monocortical values being compared. Red asterisks note a statistically significant difference between the bicortical values being compared.

Using the Shapiro–Wilk test, median absolute values for all testing showed a significant departure from normal distribution (*P*-value: 0.049). In the following paragraphs, the *P*-values are obtained by comparing the findings to the intact spine range datasets. Figure 6 plots the absolute ROM, NZ, and stiffness datasets in flexion/extension and lateral bending. The large bar indicates the median for each dataset, and the narrow bars above/under represent the interquartile range. Brackets indicate the comparison to the intact spine of the various constructs, and the asterisks indicate significant differences.

### 3.1 Range of motion

#### 3.1.1 Intact specimen

a) Flexion/extension

During the flexion/extension testing, the median ROM was 24.94 (IQR: 3.64) degrees for the six spines prepared with monocortical screws and 29.45 (IQR: 8.72) degrees for the six spines prepared with bicortical screws. Adding a unilateral monocortical and bicortical PSR, we found that the median ROM decreased (not significantly) to 24.82 (IQR: 3.19) degrees (*P* = 0.16) and increased (not significantly) to 29.93 (IQR: 8.56) degrees (*P* = 0.56), respectively. By adding a bilateral monocortical and bicortical PSR, the median ROM decreased further (not significantly) to 21.62 (IQR: 2.21) degrees (*P* = 0.003) and (not significantly) to 28.94 (IQR: 7.40) degrees (*P* = 0.06).

b) Lateral bending

During the lateral bending testing, the median ROM was 33.52 (IQR: 2.18) degrees for the six spines prepared with monocortical screws and 36.74 (IQR: 8.63) degrees for the six spines prepared with bicortical screws. By adding a unilateral monocortical and bicortical PSR, the median ROM decreased (significantly) to 24.90 (IQR: 4.79) degrees (*P* =0.003) and to 28.90 (IQR: 9.40) degrees (*P* =0.03), respectively. By adding a bilateral monocortical and bicortical PSR, the median ROM decreased (significantly) to 21.08 (IQR: 3.44) degrees (*P* = 0.003) and 22.37 (IQR: 11.24) degrees (*P* = 0.03).

#### 3.1.2 Fracture model

a) Flexion/extension

During flexion/extension testing, the bilateral monocortical and bicortical PSR median ROM significantly increased to 37.21 (IQR: 1.96) degrees (*P* = 0.003) and 42.40 (IQR: 5.60) degrees (*P* = 0.03). Following the removal of a unilateral construct, the median range of motion ROM in the unilateral monocortical and bicortical PSR fracture models significantly increased further to 44.32 degrees (IQR:4.37; *P* = 0.007) and 48.22 degrees (IQR: 3.34; *P* = 0.03), respectively. In two specimens with unilateral monocortical stabilization, screw loosening led to failure before reaching the torque threshold during testing. Supplementary Video 1 shows the behavior of a unilateral bicortical PSR construct with the vertebral body rotating around the screw shaft.

b) Lateral bending

During the lateral bending testing, the bilateral monocortical and bicortical PSR median ROM significantly increased to 23.19 (IQR: 2.75) degrees (*P* = 0.03) and 24.32 (IQR: 11.73) degrees (*P* = 0.03), respectively. The unilateral monocortical and bicortical PSR median ROM increased significantly to 42.64 (IQR: 10.88) degrees (*P* = 0.012) and 43.12 (IQR: 8.33) degrees (*P* = 0.03), respectively. In two spines with unilateral monocortical stabilization, screw loosening caused failure before reaching the torque threshold during testing.

### 3.2 Neutral zone

#### 3.2.1 Intact spine

a) Flexion/extension

During the flexion/extension testing, the median NZ was 2.39° (IQR: 0.65°) degrees for the six spines prepared with monocortical screws and 2.64° (IQR: 0.63°) degrees for the six spines prepared with bicortical screws. By adding a unilateral monocortical and bicortical PSR, the median NZ increased (significantly) to 3.90° (IQR: 1.21°) degrees (*P* = 0.03) and increased (significantly) to 4.01° (IQR: 0.76°) degrees (*P* = 0.03), respectively. By adding a bilateral monocortical and bicortical PSR, the median NZ increased (not significantly) to 2.66° (IQR: 0.73°) degrees (*P* = 0.22) and 3.13° (IQR: 0.10°) degrees (*P* = 0.06).

b) Lateral bending

During the lateral bending testing, the median NZ was 6.52° (IQR: 1.72°) degrees for the six spines prepared with monocortical screws and 6.31° (IQR: 5.46°) degrees for the six spines prepared with bicortical screws. By adding a unilateral monocortical and bicortical PSR, the median NZ decreased (not significantly) to 5.41° (IQR: 0.47°) degrees (*P* =0.06) and decreased (not significantly) to 5.86° (IQR: 2.11°) degrees (*P* = 0.56), respectively. By adding a bilateral monocortical and bicortical PSR, the median NZ decreased (significantly) to 4.65° (IQR: 2.06°) degrees (*P* = 0.03) and (not significantly) to 4.55° (IQR: 3.32) degrees (*P* = 0.09).

#### 3.2.2 Fracture model

a) Flexion/extension

During flexion/extension testing, the bilateral monocortical and bicortical PSR median NZ (significantly) increased to 9.22° (IQR: 2.37°) degrees (P = 0.03) and 7.41° (IQR: 0.86°) degrees (P = 0.03). Following the removal of a unilateral construct, the unilateral monocortical and bicortical PSR fracture model median neutral zone (not significantly) increased to 7.42° (IQR: 0.87°) degrees (P = 0.12) and significantly to 7.75° (IQR: 2.04°) degrees (P = 0.03), respectively. Two spines with unilateral monocortical stabilization reached the maximum spine tester ROM before the torque threshold testing because of failure (screw loosening).

b) Lateral bending

During the lateral bending testing, the bilateral monocortical and bicortical PSR median NZ significantly decreased to 5.36° (IQR: 0.91°) degrees (*P* = 0.03) and not significantly to 5.42° (IQR: 2.29°) degrees (*P* =0.31), respectively. The unilateral monocortical and bicortical PSR median NZ increased not significantly further to 11.99° (IQR: 4.00°) degrees (*P* = 0.12) and 7.92° (IQR: 0.87°) degrees (*P* = 0.67), respectively. Two spines with unilateral monocortical stabilization reached the maximum spine tester ROM before the torque threshold testing because of failure (screw loosening).

### 3.3 Stiffness

#### 3.3.1 Intact spine

a) Flexion/extension

During the flexion/extension testing, the median stiffness was 7.9 Ncm (IQR: 4.08 Ncm) for the six spines prepared with monocortical screws and 5.80 Ncm (IQR: 1.13 Ncm) for the six spines prepared with bicortical screws. By adding a unilateral monocortical and bicortical PSR, the median stiffness increased not significantly to 7.95 Ncm (IQR: 3.29 Ncm; *P* = 0.44) and increased significantly to 7.07 Ncm (IQR: 1.43 Ncm; *P* = 0.03), respectively. By adding a bilateral monocortical and bicortical PSR, the median stiffness increased significantly to 11.14 (IQR: 3.17) Ncm (*P* = 0.03) and 7.45 Ncm (IQR: 1.73 Ncm) degrees (*P* = 0.03).

b) Lateral bending

During the lateral bending testing, the median stiffness was 4.94 Ncm (IQR: 1.20 Ncm) for the six spines prepared with monocortical screws and 3.76 Ncm (IQR: 1.04 Ncm) for the six spines prepared with bicortical screws. By adding a unilateral monocortical and bicortical PSR, the median stiffness increased significantly to 8.00 Ncm (IQR: 0.18 Ncm; *P* = 0.03) and 7.07 Ncm (IQR: 1.89 Ncm; *P* = 0.03), respectively. By adding a bilateral monocortical and bicortical PSR, the median stiffness increased significantly to 10.68 Ncm (IQR: 2.99 Ncm; *P* = 0.03) and 9.44 Ncm (IQR: 5.23 Ncm; *P* = 0.03).

#### 3.3.2 Fracture model

a) Flexion/extension

During flexion/extension testing, the bilateral monocortical and bicortical PSR median stiffness significantly decreased to 3.14 Ncm (IQR: 0.52 Ncm; *P* = 0.03) and 3.70 Ncm (IQR: 1.15 Ncm; *P* = 0.03). Following the removal of a unilateral construct, the unilateral monocortical and bicortical PSR fracture model median stiffness did not significantly decrease to 1.49 Ncm (IQR: 0.18 Ncm; *P* = 0.12) and significantly decreased to 2.06 Ncm (IQR: 0.96 Ncm; *P* = 0.03), respectively. Two spines with unilateral monocortical stabilization reached the maximum spine tester ROM before the torque threshold testing because of failure (screw loosening).

b) Lateral bending

During the lateral bending testing, the bilateral monocortical and bicortical PSR median stiffness significantly increased to 9.18 Ncm (IQR: 2.52 Ncm; *P* = 0.03) and 7.35 Ncm (IQR: 4.17 Ncm; *P* = 0.03), respectively. The unilateral monocortical and bicortical PSR median stiffness decreased not significantly to 2.73 Ncm (IQR: 0.73 Ncm; *P* = 0.12) and 3.31 Ncm (IQR: 0.34 Ncm; *P* = 0.84), respectively. Two spines with unilateral monocortical stabilization reached the maximum spine tester ROM before the torque threshold testing because of failure (screw loosening).

## 4 Discussion

This *ex vivo* biomechanical research study aims to provide foundational insights into the biomechanics of pedicle screw-rod fixation for stabilizing the canine lumbar spine. More specifically, it compares unilateral and bilateral, monocortical and bicortical, polyaxial screw-rod fixation in a single vertebral motion unit (VMU) with the following key findings: (1) When PSR (unilateral or bilateral) are added to an intact spine, there is no significant change in the ROM in flexion/extension, whereas in lateral bending, the ROM is significantly decreased, suggesting that a single VMU PSR does not add stability to an intact spine. (2) The ROM of an intact spine was never restored using PSR (neither unilateral nor bilateral) in flexion/extension of a 3-column fracture model, suggesting that a single VMU PSR is inadequate for flexion-extension stabilization. (3) PSR failure occurred when using unilateral monocortical fixation, suggesting that this type of construct should not be used for the fractured spines (3-column affected). These *ex vivo* results led us to reject the hypothesis that a bilateral construct would be equal to or superior in rigidity to the intact spine in a fracture model. We accepted the hypothesis that a unilateral construct would not restore the rigidity of the intact spine in a fracture model.

The study's findings suggest that a one-segment construct, despite using bilateral and bicortical posterior spinal rods and fixation, is not recommended for stabilizing 3-column spinal fractures. While the addition of a bilateral bicortical or monocortical PSR increased the VMU rigidity in lateral bending compared to the intact spine, no PSR construct was able to restore the range of motion of the intact spine in flexion/extension. This contrasts with the recommendations for the management of similar injuries (AO classification B1, B2) (21) in humans, where short-segment constructs are commonly used. However, the authors note that in the human literature, if a displacement or dislocation is seen, eight implants (four bilateral monocortical pedicle screws) (22) are recommended with the implants placed in the vertebrae caudal and cranial to the fracture. The authors suspect the difference in findings between humans and dogs may be related to the method of screw placement, as in the human spine, the pedicle screws engage all three columns, whereas, in the canine lumbar spine, only two columns can be effectively stabilized (except at L7, where the pedicle is wide enough).

When assessing the neutral zone in the fracture model in flexion/extension, it was valuable to observe that bilateral PSR significantly increased NZ compared to the intact spine. This finding indicates that stability was reduced. In lateral bending, however, NZ significantly decreased, suggesting improved stability. When assessing the spine stiffness in the fracture model, the data suggests that bilateral PSR was necessary to increase stiffness. Unilateral PSR decreased stiffness in the flexion-extension plane. This may be attributed to the instability caused by the fracture, which hinders a single-sided fixation's ability to provide sufficient support in a bilateral fracture model.

The reason for the failure in the fracture models with the unilateral monocortical PSR in lateral bending and flexion/extension was a surprising finding. This suggests that the unilateral monocortical PSR construct was not strong enough to withstand the forces applied during the flexion/extension and lateral bending tests. The range of motion observed in Video 1 brings an insight into why this might have happened, as well as highlight the rotation behavior of a single vertebral body around the pedicle screw. The screws became loose, leading to the failure of the construct before the full testing protocol could be completed. Potential reasons for screw loosening with implants could include: (1) insufficient purchase/fixation of monocortical screws within the vertebral bone for the 3-column fracture model, (2) high stresses placed on the unilateral construct during the testing in this 3-column fracture model, (3) suboptimal screw placement or orientation.

To adequately oppose flexion-extension in the canine lumbar spine, the authors suggest that implants should be placed as dorsally and perpendicular to the vertebral width as possible while ensuring sufficient bone stock. Additional research is needed to evaluate the feasibility of this approach and to explore whether longer-segment constructs could enhance rigidity in flexion-extension stabilization for dogs. The orientation of the screws within the vertebral bodies (convergence vs. divergence) may also play a role and warrants further investigation. Notably, while a unilateral bicortical PSR increased rigidity compared to the intact spine, the authors could not identify why this increase was not statistically significant. Additional biomechanical studies are needed to fully elucidate the optimal stabilization strategies for 3-column spinal fractures in the canine patient.

Pedicle screw and rod systems (PSR) have become increasingly available for use in veterinary medicine. These implants possess unique characteristics that are important to understand prior to clinical application. The existing biomechanical research for veterinary PSR use has primarily focused on fixed (monoaxial) trajectory screws (15) rather than the polyaxial type examined in this study. Pedicle screws consist of a body, neck, and head. The head can be fixed in a set trajectory (monoaxial) or polyaxial. The screw body can be cylindrical or conical, with an outer and inner diameter. Generally, for human pedicle screws, a larger outer diameter, smaller inner diameter, shorter pitch, and stronger surrounding bone are thought to increase pullout strength (23). While tapping improves screw trajectory (24), it may reduce pullout strength and is therefore not recommended; underlapping by 1 mm is proposed to preserve native bone properties (25). Screw insertion can be initiated with a pilot hole, followed by pedicle probing (26) or drilling (27), with low drill speeds recommended to follow the path of least resistance. The outer diameter is the most critical factor for pullout strength, while the inner diameter primarily impacts screw fatigue. In veterinary applications, pullout strength may be less crucial than in human use, given the lack of clinically significant osteoporosis. Resistance to screw fatigue may be the more important consideration. The polyaxial head-screw coupling represents the weakest component (28). There is debate regarding the biomechanical superiority of conical vs. cylindrical inner cores, with concern that backing out conical screws could substantially reduce pullout (29). Various thread designs have been explored (30, 31), without consensus on an optimal configuration. Converging pedicle screws by 30 degrees in the coronal plane can increase pullout strength by 28% (32), though longitudinal linkage provides improved stability without convergence (33).

In the current study, 2.0 mm screws were inserted following 1.5 mm pilot hole drilling without tapping. Screw orientation and monocortical depth were not standardized.

The primary limitations of this study include the *ex vivo* nature of the testing, the small sample size, and the lack of evaluation of a non-complete fracture model. While *ex vivo* cadaveric biomechanical testing is crucial for understanding spinal biomechanics and predicting *in vivo* responses, the clinical relevance and translation to *in vivo* performance are debatable. Specifically, this study did not account for the stabilizing influence of the surrounding musculature, fascial planes, and abdominal musculature during loading and movement, which exist in live dogs. Despite previous efforts to standardize *in vitro* biomechanical testing of spinal motion segments, significant variation remains in experimental approaches (34). The small sample size of six specimens is another limitation. Prior to initiating this study, the authors reviewed the literature but did not find a clear consensus on the minimum number of samples required for an adequate biomechanical investigation (35–39). During pretesting consultation with the biomechanical spine testing laboratory (repeatability of methodology), a suggestion based on prior studies was that six specimens per group would be sufficient. Finally, the absence of testing in axial rotation represents an additional limitation of the current study.

Determining an appropriate sample size for a biomechanical study is crucial to ensure adequate statistical power and the ability to detect clinically meaningful differences. A key factor in this process is the Minimal Clinically Important Difference (MCID) (40), which represents the smallest change in an outcome measure that is considered meaningful to patients. By hypothesizing an MCID and estimating the expected standard deviation of the outcome, researchers can calculate the required sample size for a statistical test, such as a *t*-test. When the MCID is unknown, researchers can rely on Cohen's d effect size (41), representing the standardized difference between the two groups. A sample size can be calculated by specifying a desired Cohen's d and estimating the standard deviation. To determine a reasonable MCID, researchers should consult the literature to identify clinically relevant changes observed in previous studies. *Post-hoc* power analysis, which involves calculating the power of a study after data collection, is not a reliable method for assessing the adequacy of sample size (42, 43). Instead, researchers should evaluate the observed difference between groups (delta) relative to the MCID. If the delta exceeds the MCID but the statistical test is not significant, the study sample was insufficient. Conversely, the study sample was excessive if the delta is smaller than the MCID but the test is significant. By reporting the MCID and standard deviation in their research, authors can provide valuable information to future researchers for their own sample size calculations, contributing to the advancement of the field.

## Data Availability

The original contributions presented in the study are included in the article/Supplementary material, further inquiries can be directed to the corresponding author.

## Supplementary material

Supplementary Video 1 Following testing, the ROM of the VMU stabilized with unilateral bicortical PSR is demonstrated and highlights the rotation of the vertebra around the screw shaft axis in flexion/extension.
